# Mr. Eugene A. Groux—“Congenital Fissure of the Sternum”

**Published:** 1859-03

**Authors:** 


MR. EUGENE A. GROUX—"CONGENITAL FISSURE OF THE STERNUM."

We have recently had the great pleasure of examining the Singular Malformation found in the person of Mr. Groux of Hamburg, affording as it does, an exceedingly valuable and interesting Means of Studying the Motions and Sounds of the Heart and Lungs.

"M. G. is about 28 years old, small in stature, but well formed.—The remarkable peculiarity in his case consists in a fissure, which extends the whole length of the sternum at the median line, dividing it into two lateral halves. This fissure is of a V shape, with irregular outlines, covered with skin, and perhaps some of the fasciæ of the thorax; having its base upwards and its apex at the ensiform cartilage, where the two halves are held tolerably firmly together by a strong ligamentous band. During natural respiration the fissure is depressed at variable depths, which can be increased by forced respiration, giving it a concave appearance. The width of this fissure during quiet respiration is about one and a quarter inches at its upper boundary, an inch and a half upon a line with the third and fourth ribs, and a quarter of an inch at its apex. This width can be greatly diminished or increased at will by muscular effort. Through the action of the pectoral muscles, the hands joined together and pulling upon each other, the fissure is dilated to the width of about two inches and a half; while the hands being joined, and the effort being reversed—that is, pushing against each other—the fissure is lessened in width, can be closed entirely, and the edges be made to overlap even, through the influence of the deltoid and trapezii muscles. Forced expiration also increases this fissure, forced inspiration diminishes it.

Attentively examining this triangular space, a pulsating tumor is seen about its middle, on a line with the fourth rib. This is the most apparent pulsation, but there are two others in an almost vertical line with it, the one above and the other below, which can be felt; the latter is to a certain degree visible.

By forced expiration, M. Groux is able to produce a large bulging tumor in the upper portion of this fissure, which upon percussion gives the clear sound of lung. This is the upper and anterior margin of the right lung, which is forced from under the margin of the right half of the sternum to fill up the fissure, giving it a convex appearance. Coughing developes this the best.

Another curious phenomenon is the wonderful power possessed by M. Groux of instantly arresting the pulsation in the subclavian and radial arteries. This is accomplished during full inspiration, the breath being held for a few moments while the lungs are full. It is probable that the apices of the lungs press upon the subclavian arteries, and in this manner obstruct the circulation.

Still another experiment which is usually shown during the examination of this singular malformation is the enlargement of the pulsating tumor at the end of expiration, the lungs being completely exhausted, and the respiration suspended for a few moments. The tumor grows larger gradually, and the impulse of the heart is felt distinctly in the intercostal spaces of the third, fourth and fifth ribs. This is presumed to be owing to the distension of the heart by the blood during suspended respiration.

What is the pulsating tumor which is so apparent in the fissure? This is the mooted question. By some it is supposed to be the aorta, by others the right auricle, by others again the right ventricle, and by others still, the cone of the pulmonary artery or the arteria innominata."

We regret exceedingly that the rapid manner in which M. Groux is passing through the South did not permit a more extended and thorough investigation of his case, yet his demonstrations and experiments illustrating the Sounds and Motions of the Heart and Lungs, are of a deeply interesting and instructive character.

